# Cardiac manifestations of Fabry disease

**DOI:** 10.1038/s44325-025-00058-6

**Published:** 2025-08-01

**Authors:** Scott Dougherty, Dominique P. Germain, Gavin Y. Oudit, Andrew Li, Sophia Po-Yee Leung, Chen-Xu Zhao, Alex Lee

**Affiliations:** 1 Department of Cardiology, Tseung Kwan O Hospital, Hong Kong, China; 2 Division of Medical Genetics, University of Versailles-St Quentin en Yvelines (UVSQ), Paris–Saclay University, Montigny, France; 3 Division of Cardiology, Department of Medicine, University of Alberta, Edmonton, Canada; 4 Mazankowski Alberta Heart Institute, University of Alberta, Edmonton, Canada; 5 Division of Cardiology, Department of Medicine and Therapeutics, The Chinese University of Hong Kong, Hong Kong, China; 6 Laboratory of Cardiac Imaging and 3D Printing, Li Ka Shing Institute of Health Science, The Chinese University of Hong Kong, Hong Kong, China

**Keywords:** Cardiovascular biology, Cardiac hypertrophy, Cardiovascular diseases, Cardiovascular genetics

## Abstract

Fabry disease (FD, OMIM #301500) is a lysosomal disease caused by the inappropriate accumulation of globotriaosylceramide in tissues due to a functional deficiency in the enzyme α-galactosidase A. Fabry cardiomyopathy is now the most common cause of mortality in patients with FD. Large-scale metabolic and genetic screening studies have revealed FD to be more prevalent than previously thought and the later-onset variant form of FD represents an unrecognized health burden. Genetic testing is critical for the diagnosis of FD and echocardiography with strain imaging and cardiac magnetic resonance imaging using late-enhancement and T1 mapping are important imaging tools. Current therapies for FD are enzyme replacement therapy and, in patients with an amenable GLA pathogenic variant, pharmacological chaperone therapy, which can prevent FD progression, while gene therapy and the use of substrate reduction therapy represent promising novel therapies.

## Introduction

In 1898 Johannes Fabry and William Anderson both independently described Fabry disease (FD) after observing patients with ‘angiokeratoma corporis diffusum’—the red-purple maculopapular skin lesions that are now recognised as a characteristic feature of the disorder^1–3^. It was not until 1925 that the cardiac and hereditary nature of the disease were reported and it is these cardiac manifestations, characterised by structural, vascular, valvular, rhythm and conduction abnormalities and heart failure, that alongside kidney involvement and cerebrovascular disease, drive impaired quality of life and premature mortality in patients with FD^4^.

For most of its history, FD has languished as a neglected rare genetic disease. At the turn of the millennium, groundbreaking developments in the diagnosis of genetic diseases and the emergence of disease-modifying treatments have both considerably elevated interest in FD. Next-generation sequencing, leveraging the genetic information from the human genome project, has significantly enhanced our diagnostic capabilities^5^, revealing a higher burden of disease than previously thought by facilitating differentiation of FD, in particular in its later-onset form, from other causes of left ventricular hypertrophy (LVH) and other phenocopies of hypertrophic cardiomyopathy (HCM).

Enzyme replacement therapy (ERT) can prevent or slow down disease progression and irreversible organ damage^6–9^ and has sparked interest in screening for FD with the aim of early recognition and initiation of treatment. Migalastat, an oral pharmacologic chaperone and pegunigalsidase alfa, a pegylated recombinant alpha-galactosidase, were more recently approved^10,11^ and investigational therapies including gene therapy, other modified enzymes and substrate reduction therapy are on the horizon^12–15^. More recent advances in multimodality imaging, namely echocardiography with strain imaging and cardiac magnetic resonance imaging (CMR) using late-enhancement, T1 and T2 mapping, have also emerged as important imaging tools.

This paper will review these recent advances whilst also discussing expert management recommendations, which emphasise timely treatment, individualised care and multi-disciplinary input.

## Pathophysiology

FD (OMIM 301500) results from pathogenic variants in the human *GLA* gene located on the long arm of chromosome X at Xq22.1 and coding for the lysosomal enzyme α-galactosidase A (α-Gal A)^16,17^. Over 1000 *GLA* gene variants are reported^18^, categorising the disease into an early-onset, classic form associated with dramatically decreased (<3%) or absent α-Gal A activity or a later-onset (‘variant’) form in which residual α-Gal A activity (<25% in males) is present^19,20^. Due to skewed (non-random) X-chromosome inactivation, resulting in a higher proportion of X-chromosomes expressing the pathogenic variant, some heterozygous females are also at risk of manifesting any or all of the signs and symptoms of FD and of progressing to advanced disease, while random X-chromosome inactivation usually leads to an attenuated and delayed phenotype. The lack of α-Gal A activity leads to the accumulation of the enzyme’s glycosphingolipid substrate globotriaosylceramide (Gb3) and the latter’s deacylated derivative globotriaosylsphingosine (lyso-Gb3) in lysosomes and in body fluids (notably the blood).

The accumulation of Gb3 and lyso-Gb3 in lysosomes leads to lysosomal dysfunction, inflammation, oxidative stress, vascular smooth muscle cell proliferation and cell death. In turn, the latter phenomena leads to fibrosis (mainly affecting the heart and the kidneys), small vessel damage and tissue ischaemia^21^. Inflammatory and cardiac remodelling biomarkers are elevated in patients with FD consistent with heart disease leading to heart failure (usually with a left ventricular ejection fraction (LVEF) ≥ 50%)^22^.

In FD patients, histological and pathological features of the cardiovascular system include cardiomyocyte vacuolisation, concentric LVH, mid-wall fibrosis in the left ventricle, myocardial scarring, diffuse wall thickening in the coronary arteries and heart valve thickening^23–26^.

**Fig. 1:**
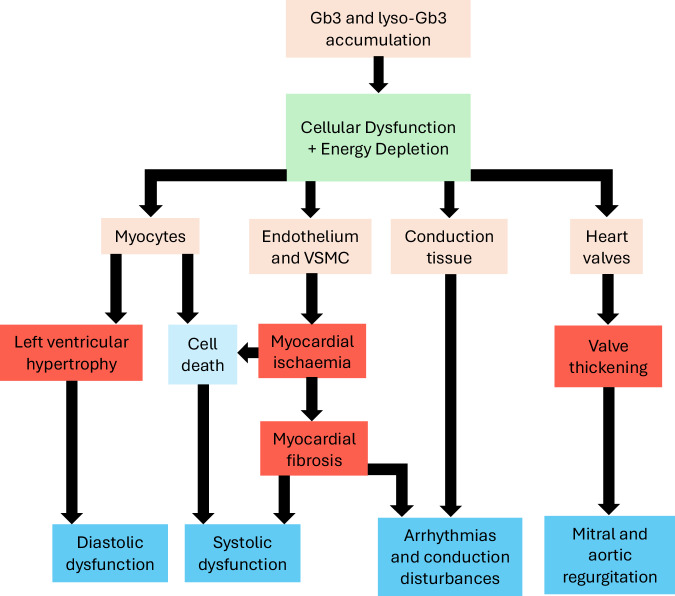
Pathophysiology of Fabry disease. Diagrammatic representation of the possible pathways between lysosomal storage and signs and symptoms of Fabry Disease. Adapted from Linhart^79^ and Pieroni et al.^48^. GB3 globotriaosylceramide, lyso-Gb3 lyso-globotriaosylceramide, VSMC vascular smooth muscle cells.

Gb3 accumulates in all cardiac cells, including cardiomyocytes, fibroblasts in the cardiac valves, the heart’s conduction system and endothelial cells^27^. The resulting endomyocardial and endothelial cell dysfunction^28^ precedes the cardiovascular signs and symptoms typical of FD (Fig. 1), making diagnosis challenging^29^. The main clinical consequence of lyso-Gb3 accumulation (rather than Gb3 accumulation) appears to be an increase in intima-media thickness^30,31^ and HCM^20^. Structural and functional changes to intramural vessels lead to myocardial ischaemia and electrical abnormalities^32,33^. Although catalytic deficiency of mutated α-Gal A is central to the pathogenesis of FD, certain *GLA* missense mutations may result in alternative pathogenic mechanisms, particularly in variant FD, including endoplasmic reticulum stress and the unfolded protein response, although external confirmation of these processes is warranted^34^.

## Epidemiology of Fabry disease

### Prevalence in the general population

While historical estimates of FD incidence were as low as 1 in 40,000 males^35^, neonatal dried bloodspot screening for α-Gal A activity deficiency in separate populations of Japanese, Austrian, Chinese-Taiwanese and Italian neonates suggest that the prevalence of FD might be higher than previously reported^36–39^. Indeed, of 21,170 Japanese neonates, 1 in 3024 were test positive; of 34,736 Austrian neonates, 1 in 3859 were test positive; of 110,027 Chinese-Taiwanese neonates, 1 in 1642 were test-positive; and of 37,104 Italian neonates, 1 in 3100 were test positive. These results suggested a hitherto unrecognised burden of FD. However, genetic testing subsequent to the dried blood spot screening programs revealed varying proportions of confirmed *GLA* pathogenic variants in newborns with α-Gal A deficiency. In the aforementioned studies, the majority of the confirmed *GLA* mutations were associated with variant phenotypes and occasionally variants of unknown significance and even (likely) benign variants. The penetrance of some *GLA* variants may be affected by the presence of systemic comorbidities, which are likely to develop later in life, such as hypertension, obesity, diabetes and smoking. It is therefore difficult to ascertain the risk posed to neonates who are diagnosed with *GLA* variants associated with non-classical disease, and long-term follow-up studies are clearly required. In any case, newborns bearing a benign or likely benign variant of GLA should not be diagnosed with FD^40^.

### Prevalence in cohorts with cardiomyopathies

A recent systematic review and meta-analysis of observational studies evaluating the prevalence of FD in patients with LVH/HCM and a recognised *GLA* variant identified 10,080 screened patients across 26 studies. Using recommendations from the American College of Medical Genetics and Genomics (ACMG), the gold standard for pathogenicity assignment, 8 variants were downgraded from pathogenic/likely pathogenic (P/LP) to variant of uncertain significance (VUS) or benign/likely benign (B/LB) (affecting 24 patients) and 4 were upgraded from VUS/B/LB to P/LP (affecting 10 patients), resulting in a pooled prevalence of FD in patients with LVH/HCM of 1.2%^41^. These results are also consistent with a cohort-based screening study in Edmonton and Hong Kong, where the prevalence of FD in patients with concentric LVH was 2%^42^.

Screening studies have also revealed that variant FD might contribute significantly to the prevalence of unexplained or idiopathic cardiovascular disorders. In small screening studies for late-onset HCM in Japanese and UK males, FD was detected in 3% and 6.3% of patients, respectively^43,44^. Furthermore in a large consecutive cohort of 1386 European patients with unexplained LVH, 0.5% had *GLA* mutations associated with FD^45^. All together, these data suggest that the prevalence of variant FD is higher than previously predicted.

However, some degree of caution must be exercised as the penetrance of mutations associated with variant FD can vary considerably. A recent review by van der Tol et al. suggests that new diagnostic algorithms are necessary to ensure that patients with FD are appropriately recognised including ruling out FD in ambiguous cases of low α-Gal A activity^46^. Moreover, whilst Fabry cardiomyopathy can be clinically indistinguishable from other genetic hypertrophic cardiomyopathies, suggesting that systematic mutation screening is necessary to better define FD patients^47^, it is important to remember that several *GLA* variants have been reclassified over the last decades from P/LP to VUS or B/LB and that there is significant discrepancy in the assignment of pathogenicity between ACMG and ClinVar, a public database commonly used in clinical practice^41^. Furthermore, the ACMG criteria has occasionally been challenged by genetic experts in the *GLA* gene^30^.

## Clinical manifestations

Cardiac involvement is highly prevalent and affects >80% of patients with FD; it includes progressive LVH, heart failure (HF), atrial fibrillation (AF), angina, non-sustained ventricular tachycardia and other arrhythmias, syncope and chronotropic incompetence (see Fig. 1)^48,49^. There is, however, considerable variation in the clinical spectrum of FD, which is dependent on gender, pathogenic variant and degree of lyonization (in females) and ranges from severe early-onset disease in males (classic FD) to asymptomatic females bearing a GLA variant associated with the later-onset phenotype – and a variety of presentations in between^50,51^.

### Classic Fabry disease

This is the most severe clinical phenotype of FD and typically affects males, although some heterozygous females can manifest full classic expression of the disease due to unfavourable lyonization. Patients with classic FD typically develop early-onset symptoms from 4 to 8 years old. These patients have very low or absent α-Gal A activity, resulting in non-specific signs and symptoms that are often mistakenly attributed to alternative disorders, leading to missed or delayed diagnoses, by which time end-organ damage may be irreversible^52,53^. Life expectancy is usually reduced, with a mean age at death of ~41 years.

The major manifestations of classical FD are cardiac, renal and neurological and these can be partially attributed to vascular endothelial dysfunction^53,54^. More than 80% of classic FD patients will develop cardiac manifestations, which usually present between 20 and 40 years of age, although heterozygous females typically present 10 years or later compared to males^55^. Cerebrovascular manifestations include early stroke and transient ischaemic attacks, which can lead to significant neurological deterioration and death^56^. Renal involvement may progress to the point of requiring dialysis or renal transplantation^57,58^.

According to the organs involved, a number of additional symptoms and signs can be found, including pain and neuropathy in the distal limbs (acroparesthesias); gastrointestinal signs and symptoms, such as diarrhoea, abdominal pain and early satiety; benign cutaneous lesions of small blood vessels (angiokeratomas); and corneal opacities (so-called cornea verticillata)^59–62^. Peripheral neurological manifestations often present earliest, but these symptoms are not definitive due to their transient and variable nature^4,52^.

### Late-onset (variant) Fabry disease

Those with variant FD typically manifest symptoms from the 4th to the 7th decades and age at death is typically >60 years old^63^. Variant FD is usually dominated by cardiac involvement, and typically lacks the systemic disease manifestations that characterise classical FD. These patients are usually diagnosed following evaluation for cardiomegaly, stroke, or proteinuria and may not have a known family history of FD^44,48,52,64^.

### Cardiovascular complications of Fabry disease

Although unexplained LVH is the well-recognised imaging hallmark of cardiac FD^65^ the clinical sequelae of FD remains under-recognised and is an important cause of HF with an LVEF ≥ 50% and ventricular arrhythmias in men aged >40 years old and women aged >50 years old^66^. Indeed, diastolic dysfunction can precede LVH and occur early in the FD disease course^67,68^, although HF with an LVEF ≥ 50% in FD patients should not be labelled as heart failure with preserved ejection fraction (‘HFpEF’), given that its pathophysiology, natural history and treatments differ to typical HFpEF patients^69^. In a FD registry of 2869 patients, HF was the most common first cardiovascular event. Overall, 5.8% of males and 3.7% of females at mean ages of 45 and 54 years, respectively, experienced cardiovascular events, which also included myocardial infarction and cardiac death^70^.

#### Ventricular hypertrophy

In a screen of FD patients, left ventricular mass index inversely correlated to α-Gal A activity, which suggests that the extent of Gb3 accumulation determines the relative extent of pathogenesis in FD^71^. Unlike male patients, myocardial functional decline and fibrosis do not always coincide with LVH in female patients with FD^72–74^. There is a strong correlation between age and the severity of LVH and all female FD carriers older than 45 years have LVH^75^.

Myocyte involvement results in progressive LVH and fibrosis, diastolic dysfunction and elevated filling pressures, leading to HF. In a minority of cases—usually advanced FD—patients may present with reduced ejection fraction. In adults, ECG signs of LVH are present in up to 61% of men and 18% of women^76^. In addition, the high prevalence of obesity and hypertension as a pan-ethnic health burden, coupled with presently long average life expectancy, can further exacerbate variant forms of FD, thereby contributing significantly to the incidence of HF^77,78^.

Concentric LVH is the most common pattern of hypertrophy observed, although asymmetric and eccentric remodelling may also occur^79^. Up to 5% of FD patients may develop asymmetric septal hypertrophy and although patients may also develop reduced LV cavity sizes and papillary muscle hypertrophy, which can lead to exercise intolerance, dynamic left ventricular outflow tract obstruction (LVOTO) appears to be rare, affecting <1% of patients^43,80^. More than one third of FD patients have right ventricular involvement, although this rarely complicates the disease course^81^.

#### Arteriopathy

The accumulation of GB3 in the walls of the arterial tree results in abnormally increased intima-medial thickness, resulting from hypertrophy and hyperplasia of the vascular smooth muscle cells^30,31,82–85^, which occurs independently of hypertension, intramural inflammation and atherosclerosis^86^.

Angina is a frequent symptom in FD patients, affecting women and men roughly equally (22% vs 23%, respectively) in an international FD outcome survey^48^. Although epicardial coronary artery disease can occur, microvascular dysfunction is typically the central underlying mechanism and replacement myocardial fibrosis is often found at areas of severe small vessel disease^87^. Vessel dilation is also commonly seen, particularly in the basilar trunk and ascending aorta and previous studies have demonstrated ascending aortic dilation in ~30–56% of males and 21% of females^76,88–90^. Aortic root dilation is usually not severe enough to require valve or root replacement.

#### Conduction disorders and arrhythmias

**Fig. 2:**
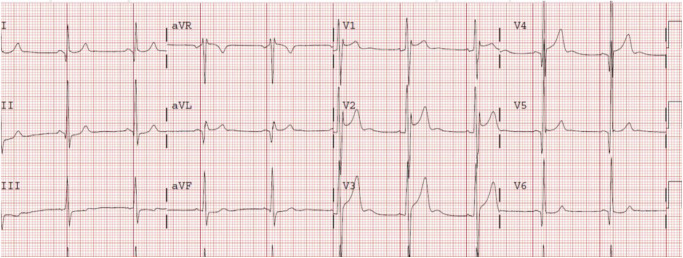
Typical ECG findings in Fabry disease. This ECG shows a shortened PR interval, LVH by Sololow-Lyon criteria and LV strain pattern. Reproduced with permission from Linhart A^81^. ECG electrocardiogram, LVH left ventricular hypertrophy, LV left ventricular hyertrophy.

Both atrial and ventricular arrhythmias occur in FD^91^. Myocardial fibrosis is associated with malignant ventricular arrhythmias (annual incidence 27%) and sudden cardiac death (annual incidence 5%) in FD patients, whilst patients without late gadolinium enhancement (LGE) did not experience malignant ventricular arrhythmias^68^. AF is common in FD, affecting males more commonly than females^91^. In younger patients, the PR interval may be shorter due to accelerated atrioventricular conduction (Fig. 2), whereas older patients may develop PR prolongation and bundle branch block (left anterior fascicular block and right bundle branch block)^92^. Complete heart block is observed in 12.7% of males and 1.6% of females in variant FD with cardiac involvement^92^.

#### Valvulopathy

**Fig. 3:**
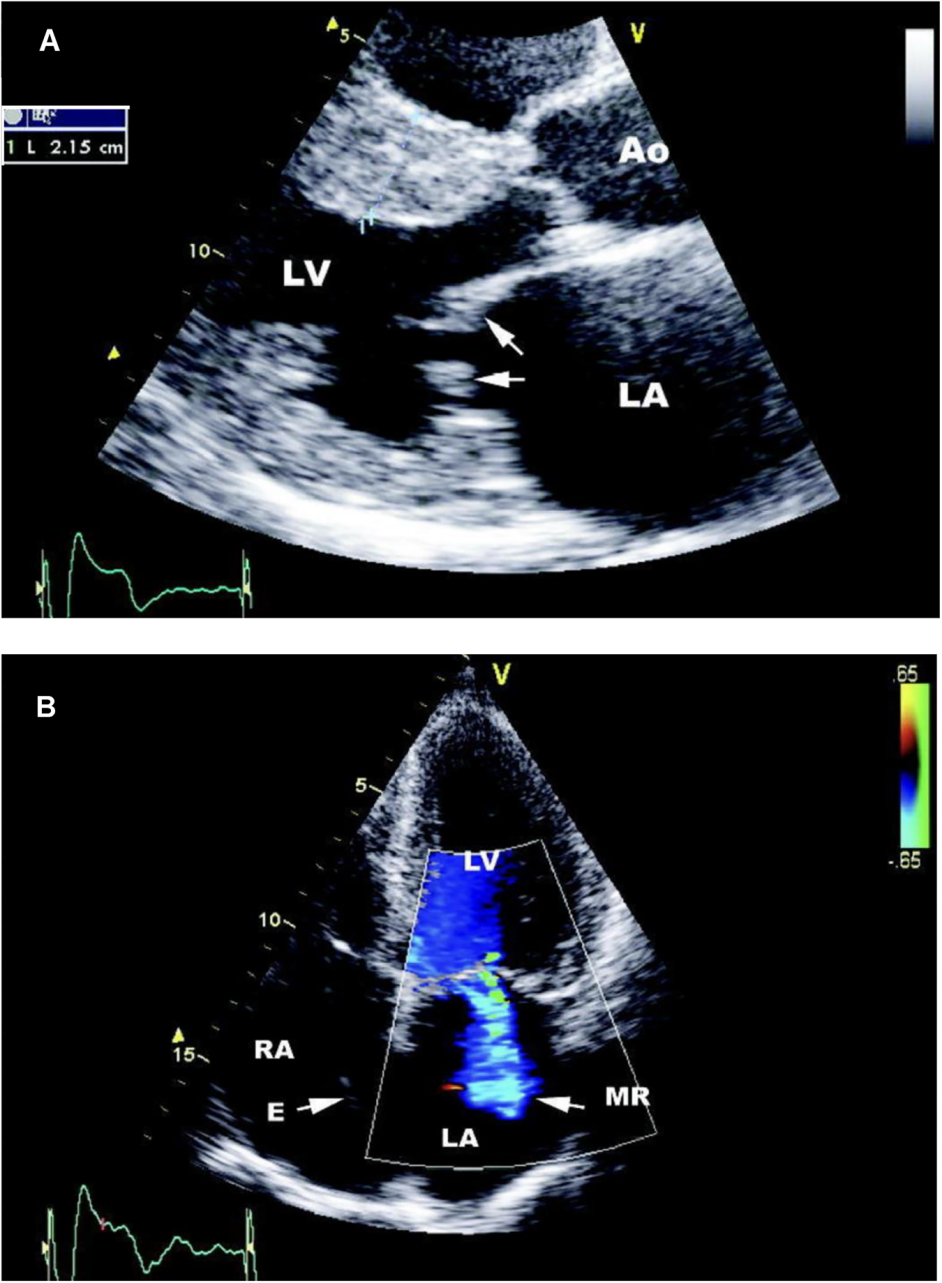
Echocardiographic findings in Fabry disease. **A** Echocardiogram showing marked interventricular sepal thickening (2.15 cm) and thickening of the MV (white arrows); **B** Echocardiogram with colour Doppler showing mild-moderate MR. Reproduced with permission from Linhart^81^. MV mitral valve, MR mitral regurgitation, LA left atrium, RA right atrium.

The left-sided heart valves are predominantly affected in FD, even though the pathological accumulation of Gb3 is present in both sides of the heart. This could be explained by the increased pressure in the left heart leading to a more rapid deterioration of the mitral and aortic valves^81,93^. Mitral leaflet thickening, with corresponding moderate or severe mitral regurgitation, is typically present in more than 50% of patients, while minor aortic valve thickening is found in nearly 50% of patients, which can progress to moderate or severe regurgitation (Fig. 3A, B)^26,71,76^.

## Diagnosis

Cardiac manifestations are the presenting symptoms in only 10–13% of patients with FD and usually only present from the third decade onwards^70,94,95^, whilst patients younger than 20 years are unlikely to manifest severe LVH (>15 mm)^49^. The non-specific signs, symptoms and complex multi-organ nature of the disease make it difficult to clinically suspect the disease without prior known family history. However, given that newer therapies may favourably alter the disease course, early diagnosis remains a vital goal.

**Fig. 4:**
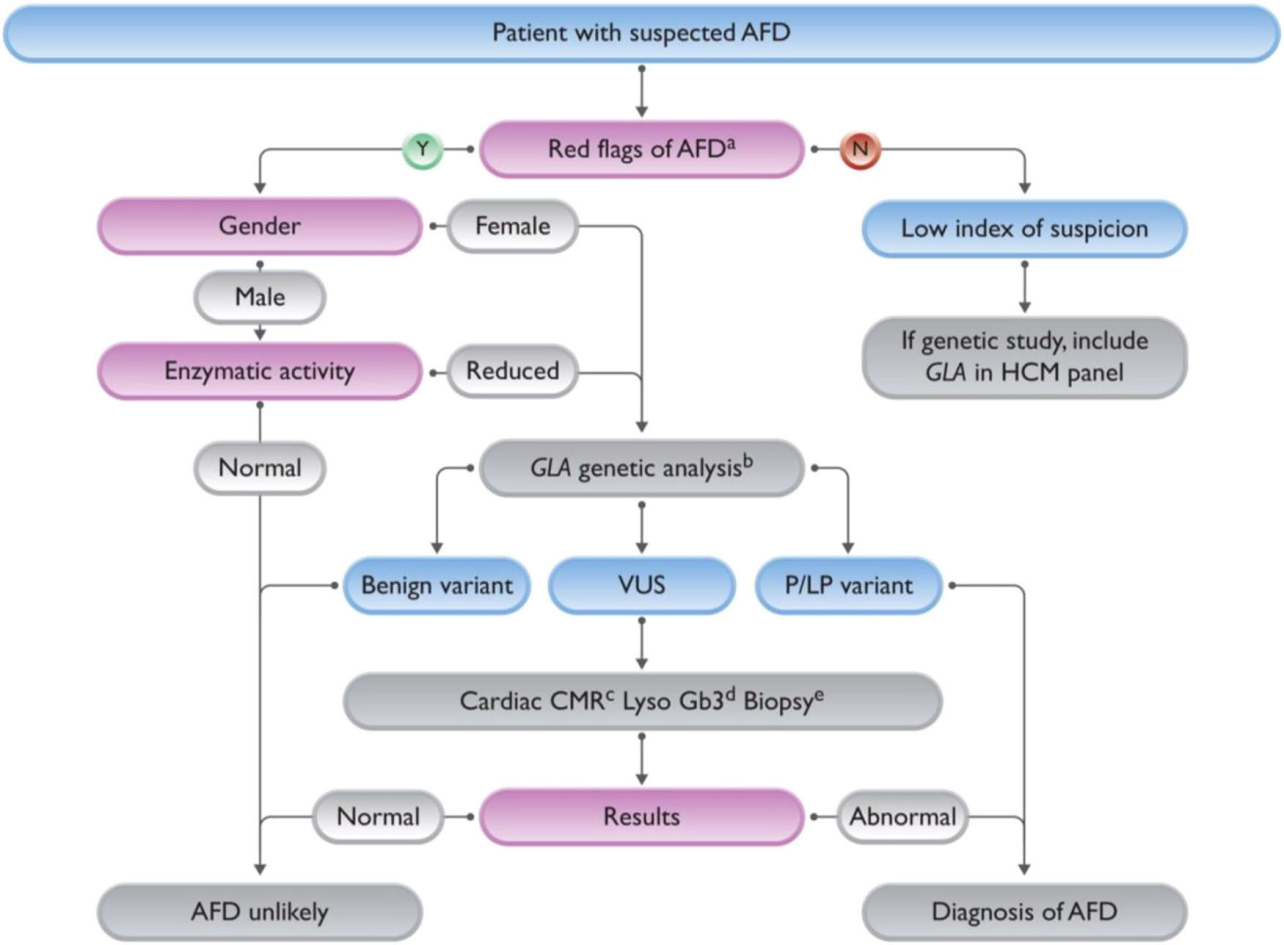
Fabry disease diagnostic algorithm. ^a^See Table 1. ^b^Genetic analysis must include the study of possible large deletions or a copy number variation not detected by the Sanger technique. ^c^The finding of increased plasma and/or urinary Gb3, or plasma lyso Gb3 and its analogues in the evaluation of male or female patients with a VUS and normal (in female patients) or lowered α-Gal A activity provides additional diagnostic information, but the role of biomarkers in such patients still requires validation. ^d^Low native T1 values reinforce or generate suspicion of Fabry disease. Normal native T1 values do not exclude Fabry disease, as they are rarely observed in untreated patients with mild LVH (mostly females), or in advanced disease due to pseudonormalization. ^e^An endomyocardial biopsy is recommended, but could be done in other affected organs such as the kidneys and skin. It should be evaluated by expert pathologists and always include electron microscopy studies to detect lamellar bodies and intracellular inclusions. Of note, some drugs may produce drug-induced phospholipidosis with an intracellular accumulation of phospholipids in different organs that can mimic zebra bodies on electron microscopy. α-Gal A alpha-galactosidase A, AFD Anderson–Fabry disease, CMR cardiac magnetic resonance, Gb3 globotriaosylceramide, HCM hypertrophic cardiomyopathy, LVH left ventricular hypertrophy, lyso Gb3 globotriaosylsphingosine, P/LP pathogenic/likely pathogenic, VUS variant of unknown significance. Reproduced with permission from Arbelo et al.^96^.

Once a patient with suspected FD is identified, diagnosis is established after careful evaluation of FD red flags, assessment of α-Gal A activity and lyso-Gb3 measurement (in male patients), *GLA* genetic testing (all female patients and male patients with reduced enzymatic activity), and advanced cardiac imaging, as summarised in Fig. 4^96^ and discussed in the following sections.

### When to suspect cardiac Fabry disease

FD should be suspected in any patient with LVH or cardiomyopathy, particularly HCM and restrictive cardiomyopathy, even in the presence of comorbidities such as hypertension or aortic stenosis because FD can co-exist with these conditions^97^. Moreover, FD accounts for ~0.3–5% of patients previously labelled as HCM, therefore it is vital to maintain a high index of suspicion and exclude FD in all HCM cases^43,98,99^. In addition to LVH, presence of unexplained HF with an LVEF ≥ 50%, ventricular arrhythmias and cardiac and extracardiac red flags (Table 1)^96^, with variable sensitivity and specificity, may further increase the suspicion for FD.

**Table 1:** Fabry disease red flags

Extracardiac red flags	
No male-to-male transmission in pedigree	
Renal involvement (dialysis, renal transplantation) or LVH in relatives	
Neuropathic pain	
Angiokeratomas	
Albuminuria	
Cornea verticillata	
Hypohydrosis, heat/cold and exercise intolerance	
Gastrointestinal symptoms (nausea, vomiting, non-specific abdominal pain, constipation, diarrhoea)	
Hearing loss (either progressive or sudden), tinnitus, vertigo	

Cardiac red flags	
ECG	Short PQ interval in young patients
	Atroventricular block in adult patients
	Bradycardia
	Chronotropic incompetence
	LVH
Echocardiogram	LVH with normal systolic function
	Hypertrophy of papillary muscles
	Mitral and aortic valve thickening with mild-to-moderate regurgitation
	Reduced global longitudinal strain
CMR	Basal-inferolateral late gadolinium enhancement
	Low native T1 (caution with ‘pseudonormalization’ in areas affected by fibrosis)
	High focal/global T2
Laboratory	Elevated high-sensitivity troponin
	Elevated NT-proBNP

Reproduced with permission from Arbelo et al.^96^.

*CMR* cardiac magnetic resonance, *ECG* electrocardiogram, *LVH* left ventricular hypertrophy, *NT-proBNP* N-terminal pro-brain natriuretic peptide.

### Diagnostic and genetic testing: importance of gender

The challenge of elucidating whether uncharacterised genetic variants are responsible for clinical manifestations of illness underscores the need for more rigorous diagnostic criteria for FD^46,100,101^. De novo mutations are rare and thus most affected males have mothers who are carriers. Traditionally, FD was diagnosed in males by markedly deficient or absent α-Gal A activity in plasma or peripheral leucocytes; however, evidence of α-Gal A pseudodeficiency suggests that confirmation of FD should be made using genetic testing when α-Gal A activity assay is ambiguous^102^.

Clinical manifestations in heterozygous female carriers range from asymptomatic to severe disease, although symptoms may initially appear mild. In a FD registry of 1077 enroled females, 69.4% had symptoms and signs of FD^55^ and the majority of female FD patients developed clinically significant disease. Carrier detection by enzyme analysis is not reliable in females, since α-Gal A activities range from very low to normal in heterozygotes and any female patient being evaluated for FD must undergo genetic testing^47^.

### Imaging evaluation of Fabry cardiomyopathy

Assessment of cardiac structural and functional abnormalities in FD can be obtained by a number of imaging modalities, including echocardiography (LVH, wall thickness, diastolic filling, valvular abnormalities) and CMR. Echocardiography has been used as a standard non-invasive screening test for Fabry cardiomyopathy for many years. However, only ~40% of patients have LVH at the time of FD diagnosis, which makes recognising cardiac involvement difficult using conventional echocardiography^76^. In addition, changes in LV diastolic function, as assessed by conventional Doppler echocardiography, have also been variable^103,104^. The challenge to conventionally diagnose FD is the significant overlap with other cardiovascular pathologies in terms of structural and functional alterations. More specificity might be achieved by way of novel approaches that exploit unique elements of the FD phenotype.

#### Echocardiography

Echocardiography can be used to assess LV remodelling and hypertrophy, estimating LV mass and assessing valvular function. New methods of strain and strain rate (SR) echocardiography have had satisfactory results for the detection of subclinical stages of impaired myocardial contractility and diastolic dysfunction in FD^105^. Tissue Doppler-derived strain and SR have been previously shown to be useful for detecting subclinical stages of impaired regional longitudinal and radial function in FD patients with otherwise normal ejection fraction^103^. In addition, peak systolic strain and SR improve with ERT, indicating that SR imaging may be a useful tool in monitoring the efficacy of treatment^6,8^.

Recent evidence shows that strain and SR analyses derived from two-dimensional speckle-tracking techniques can identify FD, independent of concentric remodelling and hypertrophy, with more sensitivity and specificity than conventional tissue Doppler echocardiography^67^. After correcting for LVH, SR during isovolumic relaxation (SR_IVR_) and transmitral E-wave velocity to SR_IVR_ ratio, remained predictors of FD by ROC analysis. Longitudinal systolic strain was also significantly lowered in FD patients compared to healthy controls^67^. Speckle-tracking imaging can provide evidence of subclinical myocardial dysfunction associated with reduced contractile reserve and diastolic dysfunction in patients with FD^106^.

#### Cardiac MRI

CMR plays a critical role in evaluating the differential diagnosis of cardiomyopathies and to characterise the structural function of the heart in patients with FD. CMR can reliably detect hypertrophy, hypokinesia and areas of delayed enhancement in patients with FD^23,107^ and is also a suitable tool for evaluating the beneficial effects of ERT on LV mass in FD patients^108^ and to delineate gender differences in LV remodelling^74,105,109^. Furthermore, CMR is sensitive to the way in which intracellular accumulation of Gb3 in FD alters the biochemical environment of the heart. It is important to note however that CMR with T1 mapping requires careful calibration and expertise, often requiring collaboration with specialised cardiac imaging centres. Collaboration with other reference centres also helps circumvent shortfalls in the availability of CMR techniques, which are not accessible in many centres. Care also needs to be made when interpreting and comparing results across different machines.

**Fig. 5:**
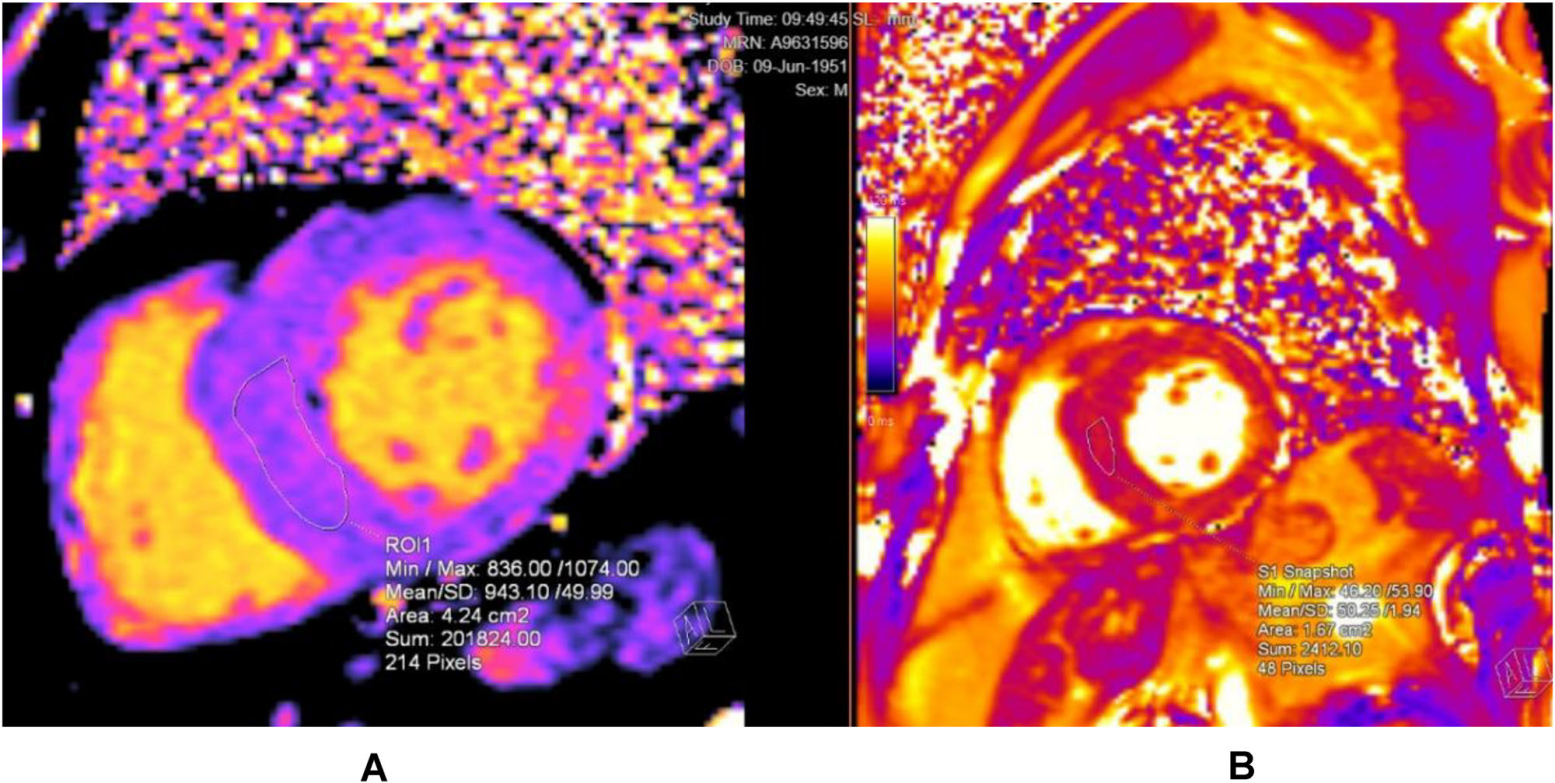
CMR assessment in Fabry disease. **A**, **B** In the T1 map on the Left, the low T1 (mean 943 ms) in the septum at 1.5 T CMR establishes the diagnosis of Fabry disease in its storage phase, indicative of sphingolipid accumulation in the septum. The T2 map on the right shows normal T2 values at the septum.

T1 mapping reveals differences in tissue pathophysiology and can elucidate biochemical differences that might not be structurally apparent, such as in idiopathic versus Fabry cardiomyopathy^110^. While FD-induced hypertrophy is structurally indistinguishable from other types of HCM, our group and Sado et al. have shown that non-contrast T1 mapping can distinguish FD from other etiologies of concentric remodelling and hypertrophy, whereby septal T1 times are significantly lower in FD patients than control or concentric remodelling patients (Fig. 5A, B and Supplementary Video 1)^74,111^. Structural analyses revealed that FD and concentric hypertrophy did not differ in terms of cardiac mass, LV chamber volume, mass to LV chamber volume ratio, or wall thickness^74^. Likewise, LVH was a common finding in diverse patient groups, including FD, hypertension and cardiac amyloidosis^111^. In both studies, T1 relaxation time was significantly lower in FD, which is likely to be a consequence of increased lipid content in the intracellular compartment of cardiomyocytes in FD^74^. Furthermore, these studies established that T1 cutoff values could be derived that separated FD from other conditions with LVH with high sensitivity and specificity^111^.

### Artificial intelligence

Formative studies evaluating the potential application of artificial intelligence (AI) in the diagnosis of FD have been encouraging, including those using medical history data alone (signs and symptoms, laboratory data, medication history, procedures and medical diagnoses)^112^, ECG analysis (with algorithms that can distinguish patients with clinical from those with subclinical cardiac involvement)^113^ and CMR analysis (distinguishing between HCM and FD)^114^.

One particularly effective AI application to echocardiography may be to help circumvent the well-known challenges of reproducing accurate measurements of LV wall thickness^115^, which clearly has significant implications for the diagnosis and treatment of FD. A machine-learning algorithm was more accurate and reproducible when measuring maximum wall thickness on a test-retest basis (patients were scanned twice on the same day) compared to 11 human experts in a large multicentre study of 60 patients with HCM (a difference of 0.7 mm vs 1.1–3.7 mm, respectively)^116^.

## Medical management

**Fig. 6:**
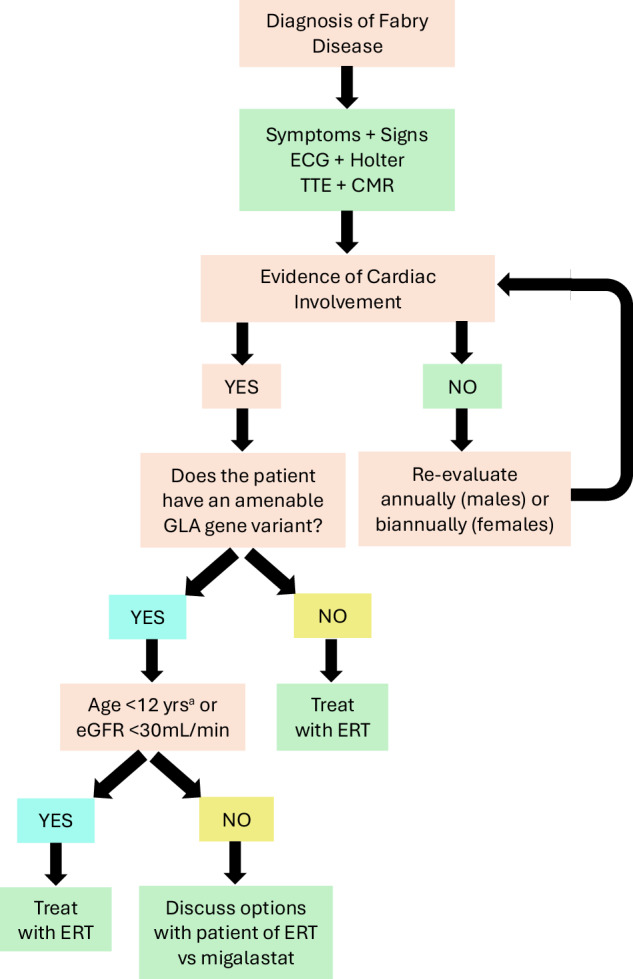
Disease-specific management of Fabry disease. ECG electrocardiogram, TTE transthoracic echocardiogram, CMR cardiac MRI, ERT enzyme replacement therapy. ^a^Migalastat is not approved for use in children <18 years old in the United States.

FD patients should be followed regularly, regardless of disease status, by a multidisciplinary team that will be able to handle the heterogeneity and variability of FD. Although signs and symptoms will dictate the frequency and extent of follow up, comprehensive medical evaluation at least once a year is recommended in all males with FD as well as females with classic phenotypes. Once a diagnosis of FD is confirmed, all individuals should have a detailed medical and family history taken. All signs and symptoms should be carefully documented at baseline and then at least annually or more often, as the clinical situation dictates. Even in the absence of symptoms, all known heterozygous females should be considered at risk for developing disease manifestations and should have a complete baseline examination. Symptomatic heterozygotes should be followed annually with tests focused on their disease manifestations, while asymptomatic females can be re-evaluated every 2 years. A summary of the approach to the initiation of disease-specific therapies is summarised in Fig. 6.

### Enzyme replacement therapy

ERT, first introduced in 2001, was the first disease-modifying therapy available for FD^117,118^ and in the decades since its establishment, clinical evidence has shown that either agalsidase-α (Replagal®, Takeda) or agalsidase-β (Fabrazyme®, Sanofi) slows, but does not reverse, the progression of cardiovascular disease in FD, with a decrease in LV mass, improved systolic and diastolic functions and a reduction in adverse cardiovascular events such as sudden cardiac death. Furthermore, ERT improves pain scores and quality of life assessments in FD patients^119^.

There is a growing body of evidence that ERT may be most beneficial in patients who have not yet developed substantial myocardial fibrosis at the time of initiation of therapy^8,120^. Indeed, among the typical features of Fabry cardiomyopathy is the development of replacement fibrosis in the basal postero-lateral segments. The fibrotic process starts in the mid-myocardial layers and spreads with disease progression towards transmural fibrosis. Thus, end-stage Fabry cardiomyopathy is characterised by the co-existence LVH and myocardial thinning.

The observed decrease in left ventricular mass among older untreated men could reflect an even more advanced stage of myocardial fibrosis. In the late stages of transmural fibrosis associated with LVH, cardiomyocyte death can induce scar tissue and thinning of the left-ventricular posterolateral wall^121^ that may be misinterpreted as a therapeutic response to enzyme replacement or chaperone therapy^73^.

The 5-year follow-up data on 181 adults (126 men) in the Fabry Outcome Survey showed that ERT with agalsidase-α was associated with (i) a significant reduction in LV mass and a significant increase in midwall fractional shortening in patients with cardiac hypertrophy at baseline, and (ii) stable values for these variables in patients without cardiac hypertrophy at baseline^122,123^. However, myocardial Gb3 content did not significantly decrease on endomyocardial biopsies taken at 6 months of treatment with agalsidase alfa^124^.

In a longitudinal analysis of data from the Fabry Registry, male patients with FD treated with agalsidase-β (1 mg/kg/2 weeks) for at least 2 years were compared with untreated men with FD^73^. When considering individuals in whom ERT was initiated between the ages of 18 and 30 (n = 31), the mean change in LV mass was −3.6 g/year (i.e. a reduction); this contrasted with a mean gain (a change of +9.5 g/year) in untreated men in the same age group. Initiation of ERT at later ages was still associated with annual gains in LV mass but these were smaller than in untreated men of the same age^73^.

In a Cochrane review of 77 cohort studies involving 15,305 participants, the pooled proportions were as follows for cardiovascular complications: agalsidase alfa 28% [95% CI 0.07, 0.55; I2 = 96.7%, *p* < 0.0001]; agalsidase beta 7% [95% CI 0.05, 0.08; I2 = not applicable]; and untreated patients 26.2% [95% CI 0.149, 0.394; I2 = 98.8%, *p* < 0.0001]. Effect differences favoured agalsidase beta compared to untreated patients^125^. Data on cardiac outcomes and benefits for the newly approved pegunigalsidase alfa enzyme, which exhibits improved pharmacokinetics parameters, are still scarce^11^.

Although ERT has cardiac benefits in both sexes, the heterogeneity of disease severity in women means that the data are less robust in these patients^55^. The evaluation of ERT efficacy in the later-onset forms of the disease with predominant cardiac involvement has been limited to a few studies in the IVS4 + 919 G > A pathogenic variant, highly prevalent in several Asian countries. In those individuals, a significant negative correlation has been published between ERT duration and Gb3 storage in cardiomyocytes and cardiomyocyte size.

Initiation of ERT should follow a multi-modality assessment. However, the potential inadequacy of ERT in some FD patients, whereby disease progression is not appreciably arrested coupled with the high annual cost of ERT (~$200,000 USD) creates a need for novel alternatives to ERT or possible adjuvant therapies for the existing ERT regimen^126,127^. Moreover, inadequate distribution of administered enzyme to various organs and tissues due to inequities in flow dynamics and receptor distribution in various tissues may limit ERT effectiveness^128^. Finally, in vitro evidence has shown that ERT-induced Gb3 clearance may not fully abolish dysregulated autophagy and fibrosis in podocytes, suggesting that Gb3-independent pathways may be implicated in FD pathophysiology^129^, and although this hypothesis that has been supported by others^130^, further research is warranted.

### New directions: chaperone therapy and gene therapy

Competitive α-Gal A inhibitors, or pharmacological chaperones, represent a paradoxical target for new therapies, whereby inhibitor molecules interact with and allow proper folding of otherwise misfolded, unstable α-Gal A variants^131,132^.

Migalastat is a first-in-class, orally administered chaperone that has been authorised by the European Medicines Agency for the treatment of FD in individuals aged 12 and over with an eGFR ≥30 mL/min/1.73 m^2^ and an amenable *GLA* pathogenic variant^133^. In a controlled trial of 67 previously ERT-(pseudo)naïve patients who started on migalastat, the mean LV mass index fell over the following 18 or 24 months of treatment (giving a mean [95% confidence interval] decrease of 7.7 [−15.4 to −0.01] g/m^2^)^10^. Migalastat was also effective on cardiac geometry (LV mass index and the interventricular septum thickness) in ERT-experienced patients who switch to migalastat^134,135^. Improvements in the LV mass and other variables (notably LGE on CMR, and blood troponin and NT-proBNP levels) have been suggested in various case series, observational studies and clinical trials^136–141^. Substrate reduction therapies^142^ and gene therapies are currently in development for the treatment of FD.

### Risk factor management and supportive care

Cardiovascular risk factor modification and treatment of HF, angina, arrhythmias and conduction disorders generally follow standard guideline recommendations, with a few important caveats that are summarised in Table 2.

**Table 2:** Risk factor management and supportive care in Fabry disease

Indication	Recommendations and Therapies
Cardiovascular Risk Factor Modification	- Smoking cessation - Dietary modification (e.g. DASH diet tailored for kidney disease) - 150 mins moderate-intensity exercise per week - Blood pressure control: ACE inhibitors or ARBs preferred - Dyslipidaemia control: statins are suitable for FD patients
Heart Failure	- Treat according to standard guideline recommendations - Beta-blockers should be used with caution given higher prevalence of sinus and AV node dysfunction in FD and patients should have Holter monitoring
Angina	- Important to distinguish coronary microvascular dysfunction from epicardial coronary artery disease, chronotropic incompetence and LVOTO - Treatment for microvascular angina as standard with careful monitoring of HR when using rate-control agents
Ventricular Tachycardia	- Burden of arrhythmias likely underestimated in FD patients, consider telemonitoring with an ILR as 24- or 48-h Holter recording may miss clinically relevant arrhythmias - - ICD and radiofrequency ablation use as per standard guideline recommendations
Atrial Fibrillation and Atrial Flutter	- Avoid chronic amiodarone therapy (may be used for poorly controlled acute episodes) as it can potentially inhibit the lysosomal degradation of phospholipids and drug-induced phospholipidosis has been described - Dronedarone is a possible alternative for rate/rhythm control but is contraindicated in permanent AF or symptomatic heart failure (heart failure with recent decompensation requiring hospitalisation or NYHA class IV) and eGFR <30 mL/min - Class Ic antiarrhythmics (e.g. flecainide) are contraindicated in patients with structural heart disease, heart failure, second or third-degree AV block and bundle branch block - The administration of any AV nodal blocking drugs should be used with caution as they may exacerbate pre-existing bradycardia and AV conduction abnormalities - Oral anticoagulation with a NOAC (or vitamin K antagonist if unavailable) in all patients regardless of CHADSVA score

*DASH* dietary approaches to stop hypertension, *ACE* angiotensin converting enzyme, *ARB* angiotensin receptor blocker, *FD* Fabry disease, *AV* atrioventricular, *LVOTO* left ventricular outflow tract obstruction, *HR* heart rate, *ICD* implantable cardioverter-defibrillator, *eGFR* estimated glomerular filtration rate, *NOAC* non-vitamin k antagonist oral anticoagulant, *NYHA* New York Heart Association.

## Conclusion

FD is an important metabolic disorder that can cause severe, variable disease manifestations in affected individuals. Large-scale metabolic and genetic screening studies have revealed FD to be more prevalent than historically thought in populations of diverse ethnic origins. Fabry cardiomyopathy, which is characterised by structural, valvular, vascular and conduction abnormalities, is now the most common cause of mortality in patients with FD. Since FD is an X-linked condition, women typically have a milder but still significant burden of Fabry cardiomyopathy. Genetic testing is widely available and plays a critical role in the diagnosis of patients with FD. With the advent of effective ERT and the continued advancement in diagnostic and evaluative methods and therapeutic agents, one should increasingly be able to provide an early diagnosis in the disease course and initiate treatment before organ damage becomes irreversible.

## Supplementary information

Supplemental video 1_FINAL

Fabry2-LGEcine.jpg

## Data Availability

No datasets were generated or analysed during the current study.
