# Supplementary material for: Cardiac manifestations of Fabry disease
Source: NPJ Cardiovasc Health. 2025 Aug 1;2:40. doi: 10.1038/s44325-025-00058-6 (PMC12912448; doi:10.1038/s44325-025-00058-6)
Supplement: Supplementary file 1 — Supplemental video 1_FINAL [file 44325_2025_58_MOESM1_ESM.pdf]

Supplemental video 1: CMR cine and LGE images in Fabry disease.

The CMR images demonstrate LVH (on the left) and LGE present in the lateral and anterior walls (post-contrast images on the right).

CMR cardiac magnetic resonance imaging; late-gadolinium enhancement LGE
